# Picosecond Dynamics of a Small Molecule in Its Bound State with an Intrinsically Disordered Protein

**DOI:** 10.1021/jacs.3c11614

**Published:** 2024-01-22

**Authors:** Gabriella T. Heller, Vaibhav Kumar Shukla, Angelo Miguel Figueiredo, D. Flemming Hansen

**Affiliations:** Department of Structural and Molecular Biology, Division of Biosciences, University College London, London WC1E 6BT, U.K.

## Abstract

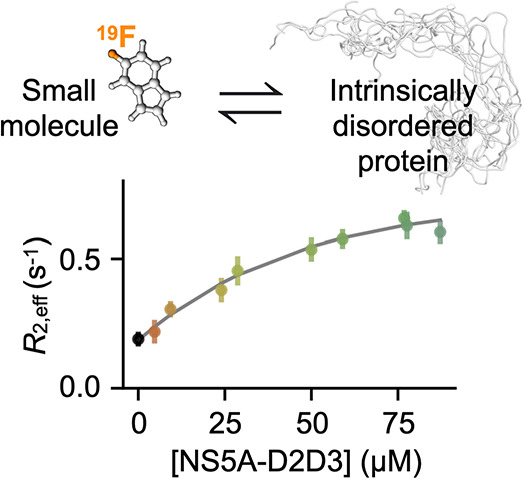

Intrinsically disordered proteins (IDPs) are highly dynamic biomolecules that rapidly interconvert among many structural conformations. These dynamic biomolecules are involved in cancers, neurodegeneration, cardiovascular illnesses, and viral infections. Despite their enormous therapeutic potential, IDPs have generally been considered undruggable because of their lack of classical long-lived binding pockets for small molecules. Currently, only a few instances are known where small molecules have been observed to interact with IDPs, and this situation is further exacerbated by the limited sensitivity of experimental techniques to detect such binding events. Here, using experimental nuclear magnetic resonance (NMR) spectroscopy ^19^F transverse spin-relaxation measurements, we discovered that a small molecule, 5-fluoroindole, interacts with the disordered domains of non-structural protein 5A from hepatitis C virus with a *K*_d_ of 260 ± 110 μM. Our analysis also allowed us to determine the rotational correlation times (τ_c_) for the free and bound states of 5-fluoroindole. In the free state, we observed a rotational correlation time of 27.0 ± 1.3 ps, whereas in the bound state, τ_c_ only increased to 46 ± 10 ps. Our findings imply that it is possible for small molecules to engage with IDPs in exceptionally dynamic ways, in sharp contrast to the rigid binding modes typically exhibited when small molecules bind to well-defined binding pockets within structured proteins.

One-third of human proteins are intrinsically disordered proteins (IDPs) that rapidly interconvert among different structures.^3−5^ Some IDPs have been suggested to interact with small molecules,^6−12^ thus opening up an enormous class of potential drug targets, especially for conditions including cancers, cardiovascular diseases, type II diabetes, and viral infections.^13^ Nevertheless, the biophysical mechanisms that underpin the binding between small molecules and IDPs are largely based on theoretical molecular dynamics (MD) simulations that, in turn, have suggested that these interactions are highly dynamic.^6,7,9,14−16^ Here, we employ experimental ^19^F-based nuclear magnetic resonance (NMR) spectroscopy to demonstrate that a small molecule remains extremely dynamic in its bound state with an IDP, in striking contrast to how most small molecules bind to structured proteins.

MD simulations have the potential to offer important insights into interactions between IDPs and small molecules; however, these calculations suffer from force field inaccuracies,^17−19^ and the reliability of these computationally expensive simulations is further hindered by long time scales required to reach convergence, especially for dynamic systems like IDPs, which undergo motions on varying time scales.^18,19^ There is also a lack of available experimental techniques suitable for characterizing interactions between IDPs and small molecules. Most of our high-resolution understanding of small-molecule/drug interactions with proteins comes from experimental X-ray crystallography, which allows scientists to resolve the atomistic details of the binding interactions, primarily enthalpic contributions including electrostatics, hydrogen bonding, π–π stacking, and hydrophobic effects. Due to their highly dynamic nature, IDPs are generally not amenable to crystallography, and given the high entropic costs associated with folding from a disordered state, it is not yet well-understood whether a small molecule could induce a single, stable structure for such experiments.

NMR spectroscopy uniquely provides atomic-resolution insights into biomolecular interactions in physiological environments,^20−22^ without the need to apply large labels nor localize the molecules on a surface—both of which may alter the structural ensemble and thus the behavior of the IDP. Standard NMR experiments, such as ligand-detected chemical shift perturbations, are commonly employed to screen and assess the binding of small molecules to structured proteins.^23^ However, chemical shifts report on the local environment of the nuclei in question, which, in turn, are averaged over time and over all the molecules in solution. Given the proposed dynamic nature of the interactions between IDPs and small-molecule ligands,^6,7,9,15^ we rationalized that NMR parameters that report on ligand dynamics and exchange might be more sensitive to detect IDP/small-molecule binding than chemical shifts.^12^ To further enhance the sensitivity of the experiment to binding, we employed ^19^F instead of ^1^H as a probe, as ^19^F has the advantage that there are no background signals in the NMR spectra from buffer components nor protein, and these spectra therefore exclusively report on the small molecule in question.

We chose the disordered domains 2 and 3 from the non-structural protein 5A (NS5A-D2D3) from the hepatitis C virus (JFH-1 genotype) as a model system, given the availability of established purification protocols and chemical shift assignments.^24−26^ Using ^19^F transverse spin-relaxation rates *R*_2,eff_ via a CPMG-based *R*_2_ experiment^1,27,28^ (Figure 1a), we identified that 5-fluoroindole (Figure 1b) interacts with NS5A-D2D3. Specifically, as the concentration of NS5A-D2D3 is increased in samples containing 50 μM 5-fluoroindole, the relaxation curves systematically decrease (Figure 1c), corresponding to an increase in the effective transverse relaxation rate of the ^19^F spin in 5-fluoroindole, *R*_2,eff_ (Figure 2a). Key features of the *R*_2,eff_ pulse program (Figure 1a) include 1) a heating compensation element to ensure that the sample is exposed to identical RF power independent of the number of CPMG blocks used^1^ and 2) an anti-ringing sequence to remove broad baseline artifacts at the on-resonance ^19^F frequency that result from sub-optimal performance of the ^1^H RF coil that has been detuned for ^19^F experiments.^1,29^ We also measured ^19^F longitudinal relaxation rates *R*_1,eff_ (Figure 2b) using an inversion recovery experiment^2^ and ^19^F chemical shift perturbations (Figure 2c) of 50 μM 5-fluoroindole in the presence of varying concentrations of NS5A-D2D3 and observed systematic changes.

**Figure 1:**
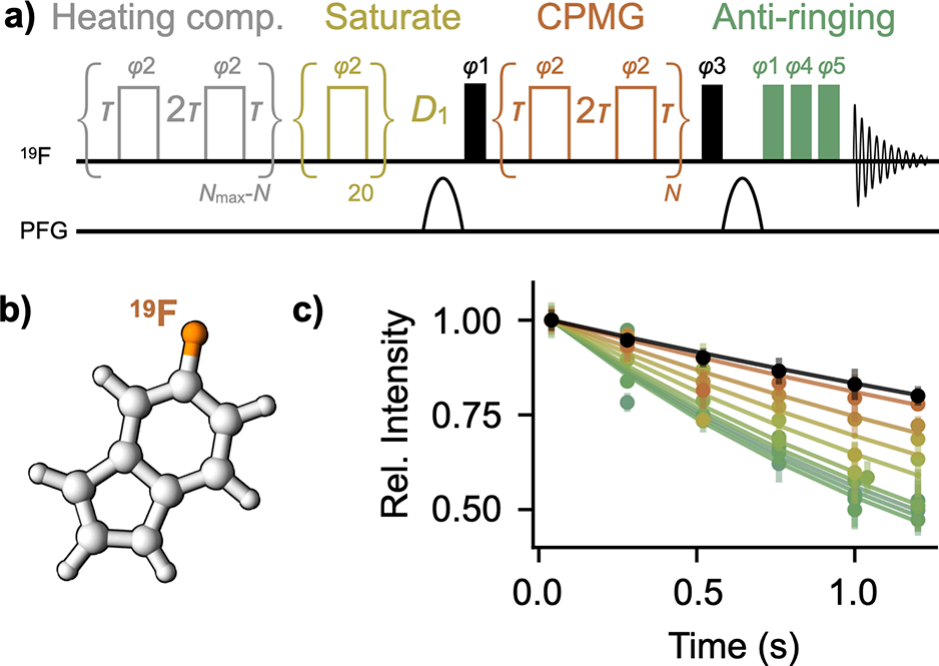
Transverse ^19^F relaxation (*R*_2,eff_) is sensitive to small-molecule binding to a disordered protein. (a) Modified pulse sequence for the ^19^F *R*_2,eff_ experiment.^1^ Narrow and wide rectangles indicate 90° and 180° pulses, respectively. The number of CPMG blocks, *N*, is varied in experiments, while the time of each block, 4τ, is held constant. (b) Structure of 5-fluoroindole. (c) Relaxation curves obtained for 50 μM 5-fluoroindole in the absence and presence of varying concentrations of NS5A-D2D3. Error bars are SEM from ≥3 technical replicates.

**Figure 2:**
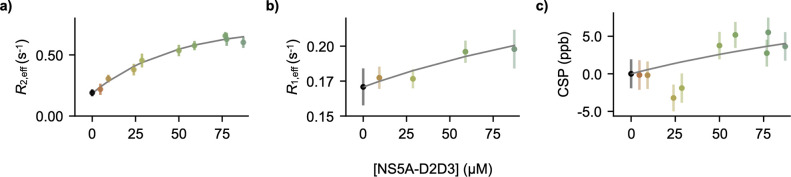
^19^F NMR reveals binding of a small molecule to a disordered protein. (a) *R*_2,eff_ rates, obtained from Figure 1c as a function of NS5A-D2D3 concentration. Error bars represent the uncertainty in the *R*_2,eff_-fitted parameter from Figure 1c (from the covariance matrix). (b) *R*_1_ relaxation rates of 5-fluoroindole measured using the ^19^F inversion recovery experiment^2^ in the absence and presence of various concentrations of NS5A-D2D3. Error bars represent the uncertainty in the *R*_1,eff_-fitted parameter (from the covariance matrix of the exponential fit to intensities from the inversion recovery experiment). (c) Quantification of ligand-detected ^19^F chemical shift perturbations relative to 50 μM 5-fluoroindole alone (Figure S1c), measured in parts per billion. Error bars represent SEM from ≥2 technical replicates. A one-site binding model (gray curve), accounting for *R*_2,eff_ rates, *R*_1,eff_ rates, chemical shifts, and translational diffusion (see text), was fit to the data, yielding an affinity constant (*K*_d_) of 260 ± 110 μM.

To gain additional insight into the interaction mechanism of 5-fluoroindole with NS5A-D2D3, a simple one-site binding model was assumed, where 5-fluoroindole can exist in one of two states: a “free” form (F) and a “bound” form (B) interacting with NS5A-D2D3. The binding mechanism is likely dynamic and more complex, but here we simply assume the “bound” form represents an ensemble of states all interacting with NS5A-D2D3. The increase in *R*_1,eff_ and *R*_2,eff_ observed with increasing concentrations of NS5A-D2D3 (Figure 2) could arise from either elevated intrinsic relaxation rates of 5-fluoroindole in the bound conformation or an exchange-induced increase in these relaxation rates.^30^ To address this, we took an integrative approach: the experimental ^19^F transverse relaxation, (Figure 2a), ^19^F longitudinal relaxation (Figure 2b), and ^19^F chemical shifts (Figure 2c, Figure S1) were combined with translational diffusion measurements of 50 μM 5-fluoroindole, measured by ^1^H diffusion ordered spectroscopy (DOSY) (Figure S2). ^1^H instead of ^19^F DOSY measurements were employed due to the relatively increased sensitivity of the ^1^H nucleus. ^19^F transverse relaxation, longitudinal relaxation, and chemical shift data involved titration of NS5A-D2D3 up to 90 μM. ^1^H DOSY measurements were performed in the absence and presence of 75 μM NS5A-D2D3. All data were analyzed simultaneously within the one-site binding model. In particular, we related free and bound longitudinal and transverse relaxation rates (*R*_1,F_, *R*_1,B_, *R*_2,F_, and *R*_2,B_) with free and bound rotational correlation times (τ_c,F_ and τ_c,B_, respectively) via well-established equations (Supporting Information).^31−34^ The free and bound rotational correlation times, the dissociation rate (*k*_off_), the dissociation constant (*K*_d_), and the diffusion coefficient of the bound form (*D*_B_) were determined from a least-squares analysis; see the Supporting Information. The diffusion coefficient of the free form of 5-fluoroindole (*D*_F_) was determined to be (1.47 ± 0.02) × 10^–9^ m^2^ s^–1^ by fitting the ^1^H DOSY data in the absence of NS5A-D2D3 (Supporting Information and Figure S2). Fits obtained using all data are shown in Figures 2 and 3. The analysis gave a *K*_d_ of 260 ± 110 μM (Figure 3a,b), a *k*_off_ of 800 ± 500 s^–1^ (Figure 3a,c), a τ_c,F_ of 27.0 ± 1.3 ps, a τ_c,B_ of 46 ± 10 ps (Figure 3b,c), and a *D*_B_ of (1.5 ± 0.6) × 10^–9^ m^2^ s^–1^.

**Figure 3:**
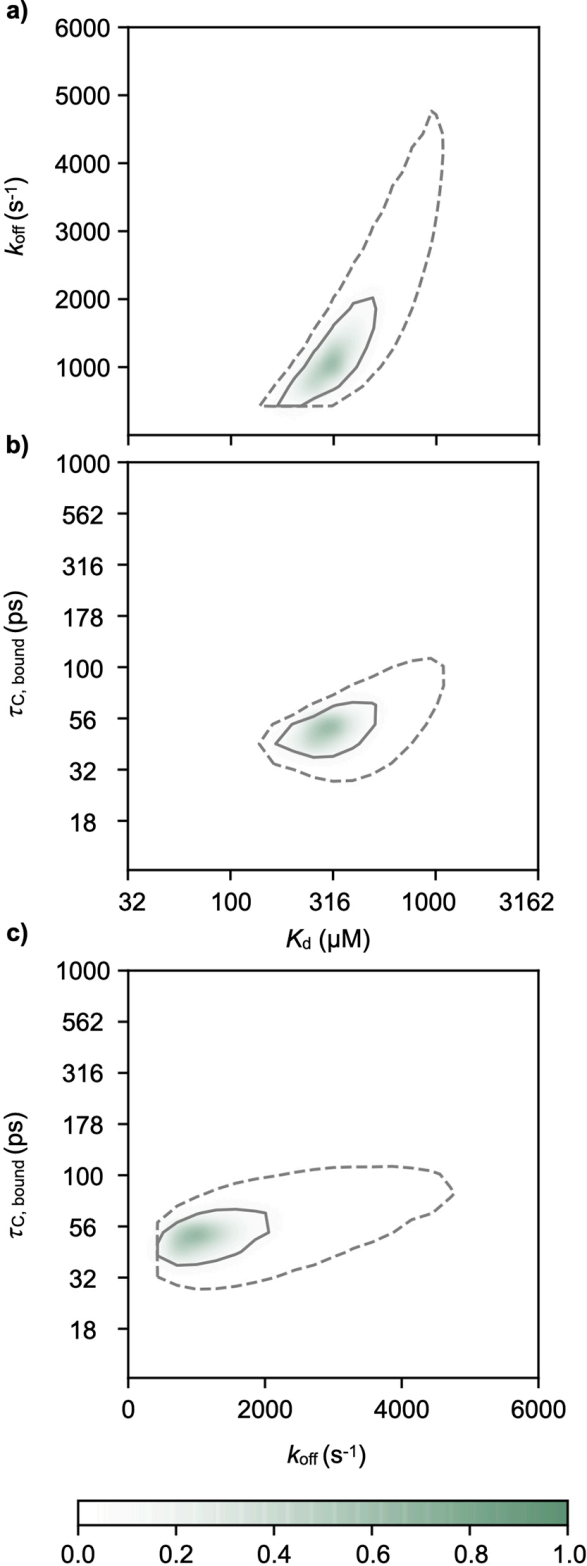
Normalized probability surfaces as functions of *K*_d_, τ_c,B_, and *k*_off_, showing a micromolar binding affinity and fast dynamics in the bound state. The surfaces shown are calculated as *p*(*x*,*y*) = exp(−χ^2^ – χ_min_^2^)/2), where χ^2^ is obtained from the least-squares fit of a one-site binding model to the experimental data. Solid and dashed lines represent the 68% and 95% confidence intervals, respectively.

The rotational correlation time of the bound state of the small molecule (τ_c,B_) of 46 ps suggests that the small molecule is extremely dynamic within the bound state. This is particularly evident when considered in the context of the rotational correlation time of typical disordered proteins. Tryptophan residues, which, like 5-fluoroindole, also contain an indole motif, within disordered protein sequences have been reported to have fast rotational correlation components between approximately 100 and 260 ps, with longer components on the low nanoseconds time scale.^35^ In this context, the τ_c,B_ that we observe, faster than the rotational correlation time of tryptophan residues in disordered proteins, suggests that the small molecule remains highly dynamic in the bound state. This extreme dynamics of the small molecule in the bound state is consistent with predictions of other small molecules interacting with IDPs.^6,9,15^

This analysis allows not only for a sensitive detection of small-molecule binding to IDPs but also the quantification of the associated dynamics of the interaction, dissociation constant, and off-rates. The value of the derived *K*_d_ is particularly of note, since the micromolar interaction observed here is the same order as often observed for lead compounds in initial drug-screening programs.^11^

To better understand the role of specificity in this interaction, we compared *R*_2,eff_ measurements of 5-fluoroindole for samples containing similar densities of either PEG-20k or 75 μM NS5A-D2D3, given that the two polymers have roughly the same molecular weight. The decrease in the relaxation profile is more significant for NS5A-D2D3 than for PEG-20k (Figure S3), suggesting that the interaction between the IDP and 5-fluoroindole is distinct from simple crowding.

It has previously been reported that, at high concentrations, both NS5A-D2 and NS5A-D3 form transient secondary structures, potentially due to the formation of multimers.^24^ To confirm that this also occurs for NS5A-D2D3, we performed circular dichroism (CD) measurements and observed a concentration-dependent loss of disorder at and above 100 μM, suggesting transient secondary structure formation (Figures S4 and S5). From the CD experiments a saturation of the more structured state could not be achieved, and therefore an equilibrium constant could not be determined for the multimerization.

Knowing that 5-fluoroindole interacts with NS5A-D2D3, we wondered whether this interaction alters the equilibrium of N55A-D2D3 related to a change in the secondary structure propensity. At NS5A-D2D3 concentrations at or below 75 μM, we observed minimal changes in the presence of 50 μM 5-fluoroindole (Figure S5a,b), consistent with the protein-detected NMR experiments (Figures S6–S8, discussed below); that is, 5-fluoroindole generally does not alter the secondary structure propensity of NS5A-D2D3. At 200 μM NS5A-D2D3, very subtle changes were observed, suggesting that 5-fluoroindole may stabilize the less disordered state adopted by NS5A-D2D3 when the protein is at higher concentrations (Figure S5c). Of particular interest is that the NS5A-D2D3 system at high concentrations provides a way to assess the binding of 5-fluoroindole to a less disordered state. Notably, when chemical shift perturbations of 5-fluoroindole were measured in the presence of 200 μM NS5A-D2D3, larger chemical shift perturbations for both ^1^H (Figure S4b) and ^19^F (Figure S4c) were detected as compared to those detected for NS5A-D2D3 in its more dilute (and thus more disordered) state. This observation coincided with an increase in ^19^F longitudinal (*R*_1,eff_) and transverse (*R*_2,eff_) relaxation rates of 0.24 ± 0.02 and 1.40 ± 0.11 s^–1^, respectively. These data suggest a further increase in the effective correlation time of 5-fluoroindole when it interacts with the less disordered state of NS5A-D2D3.

NMR chemical shift perturbations are the gold-standard technique for screening and characterizing small-molecule binding to structured proteins. While significant protein-detected chemical shift perturbations have been reported for small-molecule interactions with IDPs,^9−11^ these are often small—a fraction of the peak line widths—or nearly undetectable in cases like the one presented here (Figures S6–S8). The largest protein-detected chemical shift perturbations we observe (more than 1 SD across all residues, each less than 12 ppb), which are consistent across two concentrations of 5-fluoroindole, occur at residues A308, W312, A313, T334, S414, and S448. Furthermore, it has recently been reported that heteronuclear single-quantum coherence spectra (HSQCs) of disordered proteins are highly prone to false-positive characterization of ligand interactions due to artifacts arising from mismatched pH.^36^ In contrast, we report here that ligand-detected ^19^F transverse relaxation measurements are sensitive to small-molecule/IDP binding.

In this case study, we uncovered a micromolar binding affinity between 5-fluoroindole and NS5A-D2D3 in its disordered form, where chemical shift perturbations were minimal. We observe that transverse relaxation (*R*_2_) is more sensitive to binding than either longitudinal relaxation (*R*_1_) or chemical shift perturbations alone. In the 1D experiments, subtle changes can indeed be observed, but these could have easily been mistaken for mismatched buffers. These include very subtle increases in peak intensities, which can be explained by increases in longitudinal relaxation suggested by our model, combined with short relaxation delays (^1^H: *D*_1_ = 1 s, ^19^F: *D*_1_ = 0.5 s). We also observe subtle chemical shift perturbations in the 1D experiments. The change in chemical shift upon binding leads to a chemical-exchange contribution to the transverse relaxation but not to longitudinal relaxation, making changes upon binding easier to detect via *R*_2_-based methods as compared to *R*_1_-based experiments. We do not observe line broadening in the 1D experiments, as they are overshadowed by ^1^H–^19^F scalar couplings. Our integrative analysis, combining transverse and longitudinal relaxation, chemical shifts, and diffusion measurements, suggests that the small molecule remains very dynamic when interacting with NS5A-D2D3 in its disordered state. Our analysis of the NMR data coarsely assumes a singly averaged bound state, whereas MD simulations can frequently resolve multiple conformations within the bound ensemble.^6,7,9,37^ Additional types of NMR measurements could potentially provide further insight into the bound state ensemble.^38^

We anticipate that ^19^F ligand-detected spin-relaxation experiments offer a promising route to characterize the conformational dynamics of small molecules bound to IDPs. Furthermore, this approach could be adapted as a medium-throughput screening strategy to identify small molecules that bind IDPs and other dynamic biomolecules, especially in cases where such interactions may be largely undetectable by other approaches. Using this tool, it is feasible to quantify the binding of numerous small-molecule/IDP interactions, including point mutants. This could facilitate the exploration of crucial inquiries regarding specificity and the nature of these dynamic interactions, ultimately contributing valuable insights into the prospective “druggability” of these dynamic biomolecules.

## Data Availability

Code that supports the findings of this study is available from GitHub at https://github.com/hansenlab-ucl/R2_IDP_small_mol. All data files are available from Zenodo at https://zenodo.org/record/7892349#.ZFKHGC8w3Uo.

## Supporting Information Available

Materials, methods, ligand-detected NMR chemical shifts in the absence and presence of protein, ^1^H DOSY decays, transverse ^19^F relaxation curves of the small molecule in the absence and presence of a crowding agent, circular dichroism data of the protein at various concentrations in the presence and absence of the small molecule, and ^1^H–^15^N HSQC spectra of the protein in the presence and absence of the small molecule (PDF)

## Abbreviations

CD: circular dichroism
CPMG: Carr–Purcell–Meiboom–Gill
IDP: intrinsically disordered protein
MD: molecular dynamics
NMR: nuclear magnetic resonance
NS5A-D2D3: disordered domains 2 and 3 of the non-structural protein 5A

## References

[ref1] Overbeck J. H.; Kremer W.; Sprangers R. A suite of ^19^F based relaxation dispersion experiments to assess biomolecular motions. J. Biomol. NMR 2020, 74 (12), 753–766. 10.1007/s10858-020-00348-4. 32997265 PMC7701166

[ref2] Vold R. L. On the measurement of transverse relaxation rates in complex spin systems. J. Chem. Phys. 1972, 56 (7), 3210–3216. 10.1063/1.1677681.

[ref3] Dyson H. J.; Wright P. E. Intrinsically unstructured proteins and their functions. Nat. Rev. Mol. Cell Biol. 2005, 6 (3), 197–208. 10.1038/nrm1589. 15738986

[ref4] Csizmok V.; Follis A. V.; Kriwacki R. W.; Forman-Kay J. D. Dynamic protein interaction networks and new structural paradigms in signaling. Chem. Rev. 2016, 116 (11), 6424–6462. 10.1021/acs.chemrev.5b00548. 26922996 PMC5342629

[ref5] Tompa P. Intrinsically unstructured proteins. Trends Biochem. Sci. 2002, 27 (10), 527–533. 10.1016/S0968-0004(02)02169-2. 12368089

[ref6] Heller G. T.; Aprile F. A.; Michaels T. C.; Limbocker R.; Perni M.; Ruggeri F. S.; Mannini B.; Löhr T.; Bonomi M.; Camilloni C.; et al. Small-molecule sequestration of amyloid-β as a drug discovery strategy for Alzheimer’s disease. Sci. Adv. 2020, 6 (45), eabb5924 10.1126/sciadv.abb5924. 33148639 PMC7673680

[ref7] Heller G. T.; Aprile F. A.; Bonomi M.; Camilloni C.; De Simone A.; Vendruscolo M. Sequence specificity in the entropy-driven binding of a small molecule and a disordered peptide. J. Mol. Biol. 2017, 429 (18), 2772–2779. 10.1016/j.jmb.2017.07.016. 28743590

[ref8] Follis A. V.; Hammoudeh D. I.; Wang H.; Prochownik E. V.; Metallo S. J. Structural rationale for the coupled binding and unfolding of the c-Myc oncoprotein by small molecules. Chem. Biol. 2008, 15 (11), 1149–1155. 10.1016/j.chembiol.2008.09.011. 19022175

[ref9] Robustelli P.; Ibanez-de-Opakua A.; Campbell-Bezat C.; Giordanetto F.; Becker S.; Zweckstetter M.; Pan A. C.; Shaw D. E. Molecular basis of small-molecule binding to α-synuclein. J. Am. Chem. Soc. 2022, 144 (6), 2501–2510. 10.1021/jacs.1c07591. 35130691 PMC8855421

[ref10] Basu S.; Martinez-Cristobal P.; Pesarrodona M.; Frigolé-Vivas M.; Lewis M.; Szulc E.; Bañuelos C. A.; Sánchez-Zarzalejo C.; Bielskutė S.; Zhu J.; et al. Rational optimization of a transcription factor activation domain inhibitor. Nat. Struct. Mol. Biol. 2023, 30, 1958–1969. 10.1038/s41594-023-01159-5. 38049566 PMC10716049

[ref11] Iconaru L. I.; Ban D.; Bharatham K.; Ramanathan A.; Zhang W.; Shelat A. A.; Zuo J.; Kriwacki R. W. Discovery of small molecules that inhibit the disordered protein, p27Kip1. Sci. Rep. 2015, 5 (1), 15686 10.1038/srep15686. 26507530 PMC4623604

[ref12] Ban D.; Iconaru L. I.; Ramanathan A.; Zuo J.; Kriwacki R. W. A small molecule causes a population shift in the conformational landscape of an intrinsically disordered protein. J. Am. Chem. Soc. 2017, 139 (39), 13692–13700. 10.1021/jacs.7b01380. 28885015 PMC5962290

[ref13] Heller G. T.; Sormanni P.; Vendruscolo M. Targeting disordered proteins with small molecules using entropy. Trends Biochem. Sci. 2015, 40 (9), 491–496. 10.1016/j.tibs.2015.07.004. 26275458

[ref14] Jin F.; Yu C.; Lai L.; Liu Z. Ligand clouds around protein clouds: a scenario of ligand binding with intrinsically disordered proteins. PLoS Computat. Biol. 2013, 9 (10), e1003249 10.1371/journal.pcbi.1003249. PMC3789766 24098099

[ref15] Löhr T.; Kohlhoff K.; Heller G. T.; Camilloni C.; Vendruscolo M. A small molecule stabilizes the disordered native state of the Alzheimer’s Aβ peptide. ACS Chem. Neurosci. 2022, 13 (12), 1738–1745. 10.1021/acschemneuro.2c00116. 35649268 PMC9204762

[ref16] Thomasen F. E.; Lindorff-Larsen K. Conformational ensembles of intrinsically disordered proteins and flexible multidomain proteins. Biochem. Soc. Trans. 2022, 50 (1), 541–554. 10.1042/BST20210499. 35129612

[ref17] Rauscher S.; Gapsys V.; Gajda M. J.; Zweckstetter M.; De Groot B. L.; Grubmüller H. Structural ensembles of intrinsically disordered proteins depend strongly on force field: a comparison to experiment. J. Chem. Theory Comput. 2015, 11 (11), 5513–5524. 10.1021/acs.jctc.5b00736. 26574339

[ref18] Bonomi M.; Heller G. T.; Camilloni C.; Vendruscolo M. Principles of protein structural ensemble determination. Curr. Opin. Struct. Biol. 2017, 42, 106–116. 10.1016/j.sbi.2016.12.004. 28063280

[ref19] Bottaro S.; Lindorff-Larsen K. Biophysical experiments and biomolecular simulations: A perfect match?. Science 2018, 361 (6400), 355–360. 10.1126/science.aat4010. 30049874

[ref20] Heller G.; Yu L.; Hansen D. Characterising intrinsically disordered proteins using NMR spectroscopy and MD simulations. NMR Spectroscopy for Probing Functional Dynamics at Biological Interfaces; Royal Society of Chemistry, 2022; pp 383–410.

[ref21] Felli I. C.; Pierattelli R. Intrinsically disordered proteins studied by NMR spectroscopy; Springer, 2015.

[ref22] Shukla V. K.; Heller G. T.; Hansen D. F. Biomolecular NMR spectroscopy in the era of artificial intelligence. Structure 2023, 31 (11), 1360–1374. 10.1016/j.str.2023.09.011. 37848030

[ref23] Williamson M. P. Using chemical shift perturbation to characterise ligand binding. Prog. Nucl. Magn. Reson. Spectrosc. 2013, 73, 1–16. 10.1016/j.pnmrs.2013.02.001. 23962882

[ref24] Badillo A.; Receveur-Brechot V.; Sarrazin S.; Cantrelle F.-X.; Delolme F.; Fogeron M.-L.; Molle J.; Montserret R.; Bockmann A.; Bartenschlager R.; et al. Overall structural model of NS5A protein from hepatitis C virus and modulation by mutations confering resistance of virus replication to cyclosporin A. Biochemistry 2017, 56 (24), 3029–3048. 10.1021/acs.biochem.7b00212. 28535337

[ref25] Dujardin M.; Madan V.; Montserret R.; Ahuja P.; Huvent I.; Launay H.; Leroy A.; Bartenschlager R.; Penin F.; Lippens G.; Hanoulle X. A proline-tryptophan turn in the intrinsically disordered domain 2 of NS5A protein is essential for hepatitis C virus RNA replication. J. Biol. Chem. 2015, 290 (31), 19104–19120. 10.1074/jbc.M115.644419. 26085105 PMC4521034

[ref26] Hanoulle X.; Badillo A.; Wieruszeski J.-M.; Verdegem D.; Landrieu I.; Bartenschlager R.; Penin F.; Lippens G. Hepatitis C virus NS5A protein is a substrate for the peptidyl-prolyl cis/trans isomerase activity of cyclophilins A and B. J. Biol. Chem. 2009, 284 (20), 13589–13601. 10.1074/jbc.M809244200. 19297321 PMC2679460

[ref27] Meiboom S.; Gill D. Modified spin-echo method for measuring nuclear relaxation times. Rev. Sci. Instrum. 1958, 29 (8), 688–691. 10.1063/1.1716296.

[ref28] Carr H. Y.; Purcell E. M. Effects of diffusion on free precession in nuclear magnetic resonance experiments. Phys. Rev. 1954, 94 (3), 630 10.1103/PhysRev.94.630.

[ref29] Gerothanassis I. Methods of avoiding the effects of acoustic ringing in pulsed Fourier transform nuclear magnetic resonance spectroscopy. Prog. Nucl. Magn. Reson. Spectrosc. 1987, 19 (3), 267–329. 10.1016/0079-6565(87)80005-5.

[ref30] McConnell H. M. Reaction rates by nuclear magnetic resonance. J. Chem. Phys. 1958, 28 (3), 430–431. 10.1063/1.1744152.

[ref31] Kroenke C. D.; Loria J. P.; Lee L. K.; Rance M.; Palmer A. G. Longitudinal and transverse ^1^H–^15^N dipolar/^15^N chemical shift anisotropy relaxation interference: unambiguous determination of rotational diffusion tensors and chemical exchange effects in biological macromolecules. J. Am. Chem. Soc. 1998, 120 (31), 7905–7915. 10.1021/ja980832l.

[ref32] Abragam A. The principles of nuclear magnetism; Oxford University Press, 1961.

[ref33] Lu M.; Ishima R.; Polenova T.; Gronenborn A. M. ^19^F NMR relaxation studies of fluorosubstituted tryptophans. J. Biomol. NMR 2019, 73 (8), 401–409. 10.1007/s10858-019-00268-y. 31435857 PMC6878660

[ref34] Lu M.; Sarkar S.; Wang M.; Kraus J.; Fritz M.; Quinn C. M.; Bai S.; Holmes S. T.; Dybowski C.; Yap G. P.; et al. ^19^F magic angle spinning NMR spectroscopy and density functional theory calculations of fluorosubstituted tryptophans: integrating experiment and theory for accurate determination of chemical shift tensors. J. Phys. Chem. B 2018, 122 (23), 6148–6155. 10.1021/acs.jpcb.8b00377. 29756776 PMC6203958

[ref35] Jain N.; Narang D.; Bhasne K.; Dalal V.; Arya S.; Bhattacharya M.; Mukhopadhyay S. Direct observation of the intrinsic backbone torsional mobility of disordered proteins. Biophys. J. 2016, 111 (4), 768–774. 10.1016/j.bpj.2016.07.023. 27558720 PMC5002086

[ref36] Pandey A. K.; Buchholz C. R.; Nathan Kochen N.; Pomerantz W. C.; Braun A. R.; Sachs J. N. pH effects can dominate chemical shift perturbations in ^1^H, ^15^N-HSQC NMR spectroscopy for studies of small molecule/α-synuclein interactions. ACS Chem. Neurosci. 2023, 14 (4), 800–808. 10.1021/acschemneuro.2c00782. 36749138 PMC10348882

[ref37] Zhu J.; Salvatella X.; Robustelli P. Small molecules targeting the disordered transactivation domain of the androgen receptor induce the formation of collapsed helical states. Nat. Commun. 2022, 13 (1), 6390 10.1038/s41467-022-34077-z. 36302916 PMC9613762

[ref38] Tiwari V. P.; Toyama Y.; De D.; Kay L. E.; Vallurupalli P. The A39G FF domain folds on a volcano-shaped free energy surface via separate pathways. Proc. Natl. Acad. Sci. U. S. A. 2021, 118 (46), e2115113118 10.1073/pnas.2115113118. 34764225 PMC8609552

